# Trends in the diffusion of robotic surgery: A retrospective observational study

**DOI:** 10.1002/rcs.1870

**Published:** 2017-11-06

**Authors:** Hani J. Marcus, Archie Hughes‐Hallett, Christopher J. Payne, Thomas P. Cundy, Dipankar Nandi, Guang‐Zhong Yang, Ara Darzi

**Affiliations:** ^1^ The Hamlyn Centre, Institute of Global Health Innovation Imperial College London UK; ^2^ Department of Neurosurgery Imperial College Healthcare NHS Trust London UK

## Abstract

**Background:**

Recent studies have suggested that the use of robotic surgery for prostatectomy has been increasing, but characterization of the diffusion of robotic surgery in other procedures has not been available.

**Methods:**

Data were analysed for the years 2006–2014 using hospital episode statistics (HES), a database of all admissions to National Health Service (NHS) hospitals in England. OPCS codes were used to determine the annual number of prostatectomy, partial nephrectomy, and total abdominal hysterectomy procedures. Concurrent OPCS codes were then used to identify whether these procedures were robotic, conventional laparoscopic or open surgery.

**Results:**

The proportion of robotic cases varied depending on the surgical procedure. Diffusion of robotic surgery was relatively rapid in prostatectomy, moderate in partial nephrectomy, and slow in total abdominal hysterectomy.

**Conclusions:**

Although high institutional cost might explain the earliest delays in diffusion, this barrier does not fully account for the different rates of diffusion among surgical procedures.

## 1 INTRODUCTION

The translation of innovative devices from the laboratory to the operating room is essential to the advancement of surgical practice.^1^ In previous bibliometric analyses it has been suggested that devices developed in collaboration with clinicians and industry are significantly more likely to result in a successful first‐in‐human study and achieve regulatory approval respectively.^2^, ^3^ The subsequent adoption of such new devices by clinicians, however, remains complex and poorly understood.^4^

Robotic surgery represents among the most important surgical innovations over the last decade.^5^ Although there is little comparative effectiveness research to support the use of robotic over conventional laparoscopic surgery, it has been suggested that robotic surgery has a shorter learning curve. The clinical corollary is that robotic surgery enables many surgeons to perform laparoscopic approaches to complex procedures, when they would otherwise resort to open surgery.

Recent studies have suggested that the use of robotic surgery for prostatectomy has been increasing,^6^ but characterization of the diffusion of robotic surgery in other procedures has not been available. We therefore describe temporal trends in the nationwide use of robotic surgery within England and contrast these with conventional laparoscopic and open procedures.

## 2 METHODS

Data were analysed for the years 2006–2014 using hospital episode statistics (HES), a database of all admissions to National Health Service (NHS) hospitals in England. OPCS Classification of Interventions and Procedures (v4.5) codes were used to determine the annual number of prostatectomy (M61.1 and M61.8), partial nephrectomy (M03.1, M03.2, M03.8 and M03.9), and total abdominal hysterectomy (Q07.4) procedures. These procedures were selected because they are the highest volume procedures performed with robot‐assistance.^7^

Concurrent OPCS codes were then used to identify whether these procedures were robotic (Y74.3, Y75.3 and Y76.5) or conventional laparoscopic surgery (Y75.1, Y75.2, Y75.4, Y75.5 and Y76.8). Procedures that were neither robotic nor conventional laparoscopic surgery were assumed to be open.

Data were analysed with SPSS version 22.0 (Illinois, USA). A logistic regression model was used, with percentage of robotic cases as the dependent variable, and surgical procedure and time elapsed since introduction as independent variables. A 2‐sided *P*‐value of <0.05 was considered statistically significant.

## 3 RESULTS

**Figure 1:**
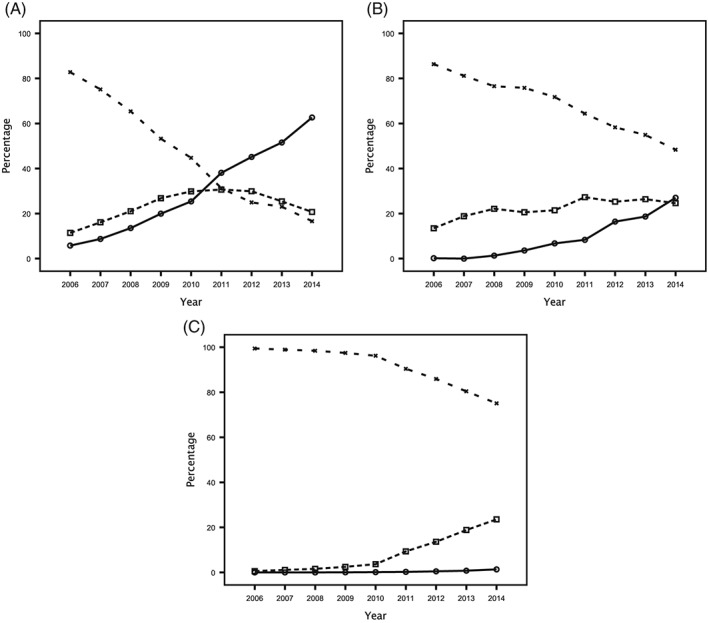
Comparison of diffusion curves for robotic procedures: (a) prostatectomy, (b) partial nephrectomy, and (c) total abdominal hysterectomy. 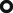 robotic 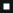 laparoscopic 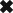 open

The number and percentages of robotic, laparoscopic, and open cases stratified by surgical procedure are shown in Tables 1, 2, 3, and Figure 1 respectively. The proportion of robotic cases varied significantly depending on the surgical procedure (*P* < 0.001), and increased significantly over time in prostatectomy, partial nephrectomy, and total abdominal hysterectomy (*P* < 0.001 in all).

**Table 1:** Annual robotic, laparoscopic, and open prostatectomy procedures performed in England (2006–2014)

Year	Total annual prostatectomy	Robotic	Laparoscopic	Open
2006–07	2537	147	290	2100
2007–08	2566	224	414	1928
2008–09	2723	369	573	1781
2009–10	3412	681	915	1816
2010–11	3614	918	1079	1617
2011–12	4176	1591	1280	1305
2012–13	4019	1814	1202	1003
2013–14	4915	2534	1249	1132
2014–15	5372	3366	1113	893

**Table 2:** Annual robotic, laparoscopic, and open partial nephrectomy procedures performed in England (2006–2014)

Year	Total annual partial nephrectomy	Robotic	Laparoscopic	Open
2006–07	534	1	72	461
2007–08	637	0	120	517
2008–09	678	9	150	519
2009–10	831	30	171	630
2010–11	1006	68	216	722
2011–12	1199	100	327	772
2012–13	1357	223	343	791
2013–14	1553	290	410	853
2014–15	1617	437	398	782

**Table 3:** Annual robotic, laparoscopic, and open total abdominal hysterectomy procedures performed in England (2006–2014)

Year	Total annual abdominal hysterectomy	Robotic	Laparoscopic	Open
2006–07	28 723	0	164	28 559
2007–08	28 094	0	309	27 785
2008–09	27 579	1	434	27 144
2009–10	27 608	13	688	26 907
2010–11	27 152	34	999	26 119
2011–12	27 335	66	2558	24 711
2012–13	26 294	127	3583	22 584
2013–14	26 894	207	5064	21 623
2014–15	27 190	368	6405	20 417

In prostatectomy, robotic surgery diffused relatively rapidly. The percentage of robotic cases increased annually, with a corresponding decrease in open cases and, in 2011, a decrease in laparoscopic cases too. By 2014, the majority of cases (62.7%) were performed robotically.

In partial nephrectomy, robotic surgery diffused at a moderate rate. Although the percentage of robotic cases increased annually, by 2014 there were a comparable proportion of robotic (27.0%) and laparoscopic (24.6%) cases, and approximately half of all cases remained open (48.4%).

In total abdominal hysterectomy, robotic surgery diffused slowly. By 2014, very few cases were performed robotically (1.4%), with the majority of cases either open (75.1%) or laparoscopic (23.6%).

## 4 DISCUSSION

### 4.1 Principal findings

Historically, all major abdominal surgical procedures were performed using open techniques. The advent of minimally invasive surgery in the 1990s was disruptive and enabled by conventional laparoscopy technology. Now, several decades later, robotic technology is speculated to stimulate a similar period of disruption as minimally invasive surgery further evolves.

The scale and pace of change that technology influences surgical practice is challenging to monitor, but made possible through large administrative databases that exist today. Interrogation of the HES database in this study has permitted quantification of the diffusion patterns for robotic surgery among its most popular applications.

In this study we have demonstrated the diffusion of robotic surgery in various procedures over time. While the trends were similar, the rate of diffusion varied considerably; diffusion was relatively rapid in prostatectomy, moderate in partial nephrectomy, and slow in total abdominal hysterectomy.

There are several factors that influence the diffusion of robotic surgery including institutional‐, surgeon‐, and patient‐specific factors. Among the greatest barriers to the adoption of robotic surgery are the high costs associated with the purchase and maintenance of such robots by healthcare institutions, particularly in publically funded healthcare systems such as the NHS.^8^ Surgeons may also be reluctant to use surgical robots that have a large operating room footprint, a prolonged setup time, lack haptic feedback, and risk malfunction or failure, particularly if such robots are not perceived to offer technical advantages over existing techniques. Finally, patients may themselves be reluctant to consent to robotic surgery.^9^, ^10^

Although high institutional cost might explain the earliest delays in diffusion, this barrier does not fully account for the different rates of diffusion among surgical procedures. We speculate that surgeon‐specific factors may instead have played an important role in explaining the findings of our study. Surgeons may find it difficult to justify use of a surgical robot when procedures have a short operating time, and are technically less complex, particularly if they are already experienced with laparoscopic techniques. In total laparoscopic hysterectomy, for example, use of the da Vinci robot takes significantly longer and does not appear to alter the conversion to laparotomy, intraoperative complications, and length of hospital stay.^11^

Patient‐specific factors may also influence adoption of robotic surgery, particularly in predominantly privately funded healthcare systems such as in the United States. It has been suggested that direct‐to‐consumer advertising has driven the incorporation of robotic surgery by competing healthcare institutions.^12^

### 4.2 Comparison with other studies

In a related study, Miller *et al*. described the temporal trends laparoscopic surgery using the Nationwide Inpatient Sample (NIS) database, a 20% nationally representative annual sample of all hospital discharges in the United States.^13^ Although the proportion of laparoscopic cases increased significantly over time (*P* < 0.001), the uptake was much more rapid in cholecystectomy and fundoplication, than hysterectomy or nephrectomy. As with the present study, these findings were thought to reflect surgeon‐ and patient‐specific factors.

### 4.3 Limitations

A limitation of this study is the use of the HES database, which does not include private cases, and may underestimate the percentage of robotic and laparoscopic cases. However, various studies have confirmed the accuracy of coding to be approximately 90%, and it is likely the key findings of this study are valid.^14^

## 5 CONCLUSIONS

The barriers to the diffusion of robotic surgery are numerous.^10^ Further research is warranted to explore the degree to which surgeon‐specific factors influence diffusion. Next generation robotic platforms, which are more customised to particular operations, may therefore better penetrate the clinical arena.

## AUTHOR CONTRIBUTIONS

HJM, AHH, CJP, and TPC were involved in the study conception, acquisition of data, analysis of data, and drafting the manuscript. DN, GZY and AD were involved in the study conception and critical revision of the manuscript.

## FINANCIAL DISCLOSURES AND CONFLICTS OF INTEREST

The authors report no conflict of interest concerning the materials or methods used in this study or the findings specified in this paper.

## DATA ACCESS, RESPONSIBILITY, AND ANALYSIS

HJM had full access to all the data in the study and takes responsibility for the integrity of the data and the accuracy of the data analysis.
